# Spatial and Temporal Profile of Glycine Betaine Accumulation in Plants Under Abiotic Stresses

**DOI:** 10.3389/fpls.2019.00230

**Published:** 2019-03-07

**Authors:** Maria Grazia Annunziata, Loredana Filomena Ciarmiello, Pasqualina Woodrow, Emilia Dell’Aversana, Petronia Carillo

**Affiliations:** ^1^Department of Metabolic Networks, Max Planck Institute of Molecular Plant Physiology, Potsdam, Germany; ^2^Dipartimento di Scienze e Tecnologie Ambientali, Biologiche e Farmaceutiche, Università degli Studi della Campania “Luigi Vanvitelli”, Caserta, Italy

**Keywords:** glycine betaine (GB), salinity, osmotic adjustment, compatible compound, CMO, ROS

## Abstract

Several halophytes and a few crop plants, including Poaceae, synthesize and accumulate glycine betaine (GB) in response to environmental constraints. GB plays an important role in osmoregulation, in fact, it is one of the main nitrogen-containing compatible osmolytes found in Poaceae. It can interplay with molecules and structures, preserving the activity of macromolecules, maintaining the integrity of membranes against stresses and scavenging ROS. Exogenous GB applications have been proven to induce the expression of genes involved in oxidative stress responses, with a restriction of ROS accumulation and lipid peroxidation in cultured tobacco cells under drought and salinity, and even stabilizing photosynthetic structures under stress. In the plant kingdom, GB is synthesized from choline by a two-step oxidation reaction. The first oxidation is catalyzed by choline monooxygenase (CMO) and the second oxidation is catalyzed by NAD+-dependent betaine aldehyde dehydrogenase. Moreover, in plants, the cytosolic enzyme, named *N*-methyltransferase, catalyzes the conversion of phosphoethanolamine to phosphocholine. However, changes in CMO expression genes under abiotic stresses have been observed. GB accumulation is ontogenetically controlled since it happens in young tissues during prolonged stress, while its degradation is generally not significant in plants. This ability of plants to accumulate high levels of GB in young tissues under abiotic stress, is independent of nitrogen (N) availability and supports the view that plant N allocation is dictated primarily to supply and protect the growing tissues, even under N limitation. Indeed, the contribution of GB to osmotic adjustment and ionic and oxidative stress defense in young tissues, is much higher than that in older ones. In this review, the biosynthesis and accumulation of GB in plants, under several abiotic stresses, were analyzed focusing on all possible roles this metabolite can play, particularly in young tissues.

## Glycine Betaine Metabolic Pathways

Diverse halophytes, but only a few crop plants, including Poaceae, synthetize and accumulate glycine betaine (GB) in response to environmental constrains (Weretilnyk et al., 1989).

**FIGURE 1:**
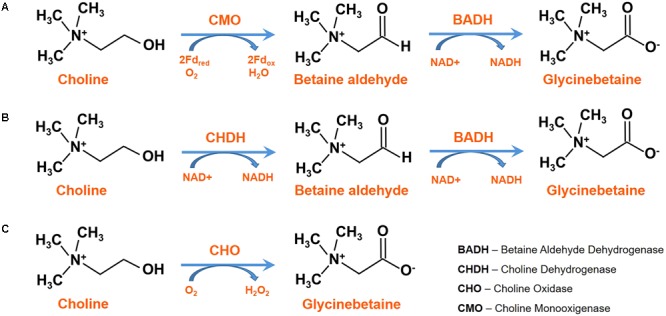
Alternative biosynthetic pathways for glycine betaine (GB) in **(A)** plants, **(B)** animals and many bacteria and **(C)** *Arthrobacter globiformis* and *Arthrobacter pascens.*

In plants, GB synthesis starts from choline, which, in turn, is synthesized through three sequential adenosyl-methionine dependent methylations of phospho-ethanolamine (PE) catalyzed by the cytosolic enzyme phospho-ethanolamine *N*-methyltransferase (PEAMT; EC 2.1.1.103) (Nuccio et al., 2000). The PEAMT enzyme has two methyltransferase domains in tandem at the N and C-terminal domains; the former converting PE into phospho-monomethylethanolamine (P-MME), and the latter methylating P-MME to phospho-dimethylethanolamine (P-DME) and P-DME to phospho-choline (Brendza et al., 2007). The product of PEAMT is phospho-choline (PC) which in different plants can undergo different pathways for the transformation to choline. In spinach, PC is directly dephosphorylated to choline, while in tobacco it is first included in phosphatidyl-choline and then metabolized to choline (McNeil et al., 2001). Subsequently, GB is synthesized by two oxidations on choline, via betaine aldehyde, catalyzed by a ferredoxin-dependent choline monooxygenase (CMO; EC 1.14.15.7), and a NAD^+^-dependent betaine aldehyde dehydrogenase (BADH; EC 1.2.1.8), respectively (Rhodes and Hanson, 1993; Sakamoto and Murata, 2002; Chen and Murata, 2011) (Figure 1A). CMO has a Rieske-type [2Fe-2S] active site, in addition to a transit peptide sequence, and it is usually localized in the chloroplast or other subcellular compartments (Rathinasabapathi et al., 1997). BADH can be either NAD^+^ or NADP^+^ dependent, but in plants it shows higher activity with NAD^+^ (Fitzgerald et al., 2009). It belongs to the superfamily of aldehyde dehydrogenases, and also has a non-specific action on other aldehyde substrates: this also explain its presence in non-GB accumulating plants or in organs of plants that do not contain GB (Rhodes et al., 2002). BADH is induced by abscisic acid (ABA) in cereals, but neither NaCl, ABA nor turgor reduction seem to be directly involved in the induction, but rather in an unknown signal coming mainly from roots as well as other plant parts (Takabe et al., 1998).

Chenopodiaceae, such as spinach and sugar beet, have CMO and BADH enzymes localized in the chloroplast stroma (Fujiwara et al., 2008). In *Hordeum vulgare*, a peroxisomal NADPH-dependent CMO is involved in the first step of GB synthesis exerting choline oxidation; while BADH is localized in the cytosol and in the chloroplast (Weigel et al., 1986).

In animals and many bacteria, a membrane-bound choline dehydrogenase (CHDH; EC 1.1.99.1) catalyzes the oxidation of choline to betaine aldehyde; while the enzyme involved in the second step is BADH again (Figure 1B). In *Arthrobacter globiformis* and *Arthrobacter pascens* a soluble choline oxidase (CHO; EC 1.1.3.17), coded by a single gene *codA*, catalyzes the direct four-electron oxidation of choline to GB, with betaine aldehyde as an intermediate. It contains a covalently linked flavin adenine dinucleotide (FAD), and acts through two hydride-transfer reactions, the two reductions of flavin and the rate-limiting steps (Ikuta et al., 1977; Rozwadowski et al., 1991; Fan and Gadda, 2005) (Figure 1C). This enzyme has become relevant due to its biotechnological applications for the metabolic engineering of economically important plants for osmotic stress resistance (Sakamoto and Murata, 2000), and the production of sensors for the determination of choline and derivatives in biological fluids (Shimomura et al., 2009; Salvi et al., 2014).

Glycine betaine can also undergo a stress-inducible synthesis, since it may be derived from the serine that is synthetized by the (i) non-phosphorylated glycerate pathway, (ii) the phosphorylated phospho-hydroxypyruvate pathway (Kleczkowski and Givan, 1988; Igamberdiev and Kleczkowski, 2018), or iii) the salt-stress-induced photorespiratory glycolate pathway (Wingler et al., 2000; Carillo et al., 2008). Serine is the precursor of PE via ethanolamine. In plants, the latter is formed directly by the action of a pyridoxal 5′′-phosphate-dependent L-serine decarboxylase (SDC) (Rontein et al., 2001); while in plants and animals, but not in fungi, it can indirectly be synthetized through a base exchange between serine and existing PE (Kwon et al., 2012). Thereafter, the phosphorylation of ethanolamine is catalyzed by a choline/ethanolamine kinase (CEK; EC 2.7.1.32).

Salinity, specifically, increases the *CMO* and *BADH* gene expression two- to three-fold and, consequently, the corresponding enzyme levels (Weretilnyk et al., 1989; Weretilnyk and Hanson, 1990; Rhodes et al., 2002).

Xu et al. (2018) identified a CGTCA-motif in the promoter region of *Citrullus lanatus CMO* and *BADH* genes that are responsive to methyl jasmonate (MeJA). When *C. lanatus* cells were activated by MeJA, they synthetized GB even without osmotic stress, and the new cells derived by the activated ones retained a high GB content without previous stress or MeJA activation. This finding suggests that JA signal transduction is involved in GB biosynthesis, which plays a key role in both osmotic stress tolerance and osmotic stress hardening (Xu et al., 2018).

However, the interactive effects of simultaneous salinity and other stresses can decrease the expression of *CMO* and the relative GB production (Carillo et al., 2011; Woodrow et al., 2017; Ciarmiello et al., 2018). This is particularly relevant because the step catalyzed by CMO is the rate-limiting one in GB biosynthesis (Bhuiyan et al., 2007). Moreover, it is important to underline that GB is not actively metabolized, therefore its concentration depends on the control of its synthesis, transport and dilution by growth (Rhodes and Hanson, 1993; Carillo et al., 2008).

Ciarmiello et al. (2018), showed that a transcript in durum wheat, coding a putative CMO-like enzyme with a different Rieske-type motif that showed similarity with the CHO, was isolated in *Ruegeria* sp., *Pseudomonas fluorescens*, and *Rhodococcu*s sp. suggesting a possible alternative pathway for the production of GB in durum wheat similar to that operating the direct oxidation of choline to GB.

## Genetically Engineered Biosynthesis of GB

Different genera, and even different species within the same genus, accumulate contrasting amounts of GB, and are therefore classified as accumulators and non-accumulators (Rhodes and Hanson, 1993). Natural accumulators of GB accumulate large amounts of GB only under abiotic stresses (Storey et al., 1977). Homozygous lines for Bet1 (GB accumulators), that are part of near-isogenic maize lines presenting different GB accumulation capacity, showed a 10–20% higher biomass under salinity than the non-accumulating lines (Saneoka et al., 1995; Munns and Tester, 2008). Therefore, metabolic engineering strategies, aimed at increasing the synthesis and accumulation of GB, have been associated with an amelioration in plant stress tolerance. In particular, important crop species like rice (*Oryza sativa*), potato (*Solanum tuberosum*), and tomato (*Solanum lycopersicum*), which are not able to synthesize and accumulate GB, have been considered as potential targets for metabolic engineering of GB biosynthesis (McCue and Hanson, 1990).

Several genes involved in the GB biosynthetic pathway have been isolated, cloned and used to generate transgenic plants, accumulating GB with an enhanced tolerance to abiotic stress (Wani et al., 2013). The transformations were quite successful for several plant species, improving plant tolerance to salt, drought and extreme temperatures, notwithstanding the very low amounts of GB accumulated by the engineered plants (Nuccio et al., 2000; Sakamoto and Murata, 2002; Chen and Murata, 2008). Among such engineered plants, those transformed with *codA* from *A. globiformis*, encoding the enzyme CHO that catalyzes the direct oxidation of choline to GB, also showed the accumulation of GB directly in the chloroplasts. In particular, a successful transformation with *codA* was obtained in *Arabidopsis thaliana* (Hayashi et al., 1997, 1998; Sulpice et al., 2003), *Brassica chinensis, Brassica juncea*, and *Brassica napus* (Huang et al., 2000; Prasad et al., 2000; Wang et al., 2010), *O. sativa* (Sakamoto and Murata, 1998; Mohanty et al., 2002; Kathuria et al., 2009) and even in woody plants such as the Japanese persimmon (Gao et al., 2000) and *Eucalyptus globulus* (Yu et al., 2009). The plants engineered with *codA* showed an enhanced tolerance to chilling, freezing, salinity, high temperature, and high light in different growth stages, from seed germination to growth, development and reproductive stages (Wani et al., 2013). Likewise, significant success has been achieved by engineering plants with *betA, betB* or both genes from *Escherichia coli* encoding CHDH and BADH, respectively, such as in *Gossypium hirsutum* (Lv et al., 2007), *Medicago sativa* (Liu et al., 2011), *Nicotiana tabacum* (Holmström et al., 2000). *codA-*transformed rice plants, showed a GB concentration of 5 and 1 μmol g^-1^ fresh weight in leaves when the transformation was targeted to the cytosol and chloroplast, respectively (Sakamoto and Murata, 1998). While, the transformation of maize, an accumulator plant, with *betA* increased GB concentration to about 5.7 μmol g^-1^ fresh weight, a value higher than that present in wild-type (WT) maize plants under drought stress (Quan et al., 2004).

However, the efficacy of GB engineering for important cultured field crops has never been demonstrated. The main reason is that even if the levels of GB in the engineered plants were significantly increased, they were still lower than those of high accumulator species, which range from about 4 to 40 μmol g^-1^ fresh weight (Rhodes and Hanson, 1993; Sakamoto and Murata, 2002). One possible explanation for this is that choline availability may limit GB accumulation in some plants. In fact, transformed tobacco plants, with a spinach cDNA encoding *CMO*, showed a very low GB production of about 0.02–0.05 μmol g^-1^ fresh weight in both control and salt stress conditions, and were able to accumulate large amounts of GB only when choline was supplemented (Nuccio et al., 1998). Moreover, the concentration of endogenous choline did not change significantly in all transgenic plants expressing the *codA* gene (Giri, 2011). Its availability does not affect the GB synthesis of all transgenic plants, most probably due to synergism in the demand and supply of choline to chloroplast.

In fact, the cytosolic choline in plants, synthetized in the cytosol or exogenously supplied, needs to be transported to the chloroplast for GB biosynthesis. Therefore, it could be possible that different capacities of plants to synthetize GB, could be also dependent on their diverse ability to transport choline to the chloroplast and not only on its availability in the cytosol (McNeil et al., 2000; Khan et al., 2009). Besides, transformed tobacco plants overexpressing CHO in the chloroplast and supplemented with choline, accumulated GB at only 1 μmol g^-1^ FW, while Arabidopsis which over expressed CHO in the cytosol and were supplemented with choline, accumulated GB at about 120 μmol g^-1^ FW (Huang et al., 2000; Fariduddin et al., 2013). Similarly, CHO-transgenic tomato plants were able to accumulate more GB in the cytosol than in the chloroplast (Park et al., 2007). CHO-transgenic maize and rice were able to accumulate similar amounts of GB in both subcellular compartments, confirming that, independent of choline concentration in the cytosol, its species-specific capacity of transport from the cytosol to the chloroplast, highly affects GB production in the chloroplast and the plants tolerance to stress (Khan et al., 2009).

Several plant species engineered to express CMO and/or BADH and which are supplemented with 10 mM betaine aldehyde, are able to synthesize GB in amounts comparable to accumulator plants (Chen and Murata, 2011). Specifically, *N. tabacum, O. sativa* and *Daucus carota*, transformed with *betB* and supplemented with betaine aldehyde, were able to produce 4.6, 6, and 10 μmol g^-1^ fresh weight GB (Kishitani et al., 2000; Kumar et al., 2004; Yang et al., 2007), demonstrating that a significant increase of GB is achievable.

Recently, a novel gene, *GB1*, differentially expressed in low and high GB accumulating genotypes of maize, was identified by Castiglioni et al. (2018). Transgenic *GB1*-maize and soybean lines accumulated GB at concentrations 4–10-fold higher than WT plants. GB1 protein is a member of the Pfam fatty acid hydroxylase superfamily, with a suggested peroxisomal location. Its predicted sequence showed 60% identity as a putative C-4 sterol methyl oxidase from rice. GB1 protein certainly has a main role in the GB accumulation in plants, and can be used as an innovative tool to improve tolerance to abiotic stress in crop plants (Castiglioni et al., 2018).

## Exogenous GB Applications

Glycine betaine improves growth and survival of plants counteracting metabolic dysfunctions caused by stress. Due to the beneficial effects of GB, numerous experiments of exogenous application of this compatible compound, on low-accumulator and non-accumulator plant species have been done. Recent studies, and related reports have proven its effectiveness in increasing plant tolerance to various stresses (Table 1). Exogenous GB is able to preserve Photosystem II (PSII) and the photosynthetic oxygen evolving complex (OEC) association under salinity in *Lycopersicon esculentum* (Mäkelä et al., 1998, 1999; Park et al., 2006), *H. vulgare* (Oukarroum et al., 2012) and *N. tabacum* (Ma X.L. et al., 2006), and to induce the expression of oxidative stress response genes, decreasing ROS accumulation and lipid peroxidation in cultured tobacco cells under salinity (Demiral and Türkan, 2004; Banu et al., 2010). Application of GB to bread wheat leaves, reduces the accumulation of Na^+^ and increases the accumulation of K^+^ and Ca^2+^, improves leaf water potential, enhances the activities of SOD, CAT and POD and, reduces photoinhibition enhancing growth and yield (Ma Q.-Q. et al., 2006; Raza et al., 2007, 2014).

**Table 1:** Effect of exogenous GB under abiotic stress conditions.

Crop	Abiotic stress	Effect of exogenous GB under abiotic stress conditions	Reference
*Brassica napus*	Osmotic stress	Inhibition of osmo-induced proline response, inhibitory effect on protein synthesis	Gibon et al., 1997
*Brassica rapa*	Drought and salt stress	Increased net photosynthesis, increased stomatal conductance, decrease of photorespiration	Mäkelä et al., 1999
*Glycine max*	Salt stress	Reduced lipid peroxidation (MDA content), increased proline content of seedlings, increased CAT and APX enzyme activity, reduced ROS level, reduced Na^+^/K^+^ ratio	Malekzadeh, 2015
*Hordeum vulgare*	Cold stress	Increase in total osmolality, higher endogenous GB levels, induction of *wcor410* and *wcor413* genes, improved tolerance to photoinhibition of PSII	Allard et al., 1998
*Hordeum vulgare*	Heat stress	Increase tolerance of PSII and protective effect on the OEC (oxygen evolving complex)	Oukarroum et al., 2012
*Lolium perenne*	Salt stress	Higher shoot and root fresh weight, lower decline of RWC and Chl, reduced electrolyte leakage and MDA content, increased GB content, SOD, CAT and APX activity, reduced Na^+^/K^+^ ratio in leaves and stems	Hu et al., 2012
*Lycopersicon esculentum*	Cold stress	Higher PSII activity, lower H_2_O_2_ levels, increased catalase activity and catalase gene (*CAT1*) expression	Park et al., 2006
*Lycopersicon esculentum*	Drought and salt stress	Increased net photosynthesis and stomatal conductance, decrease of photorespiration	Mäkelä et al., 1999
*Lycopersicon esculentum*	Salt and heat stress	Increased fruit yield, increased rate of net photosynthesis	Mäkelä et al., 1998
*Medicago sativa*	Cold stress	Reduced loss of ions from the shoot tissues	Zhao et al., 1992
*Nicotiana tabacum*	Drought stress	Improved growth of plants, improved osmotic adjustment, enhanced photosynthesis, higher efficiency of PSII, increased anti-oxidative enzyme activities	Ma et al., 2007
*Oryza sativa*	Salt stress	Improved height, fresh weight and dry weight in plant, enhanced total chlorophyll and proline content, reduced MDA content	Yao et al., 2016
*Phaseolus vulgaris*	Salt stress	Higher plant fresh weight, increased values of leaf area ratio, leaf area index, RWC and MSI (Membrane Stability Index), higher total soluble sugar and free amino acids concentrations in the leaves and pods	Osman and Salim, 2016
*Pisum sativum*	Drought stress	Enhanced growth, pods and leaves number per plant, increased level of soluble sugars, higher free amino acids and soluble proteins in leaves, increased activity of antioxidant enzymes, reduction of proline accumulation	Osman, 2015
*Prunus persica*	Cold storage	Lower content of MDA, higher level of endogenous GB, increased activity of BADH, P5CS and OAT, increased GABA content, higher level of ATP content	Shan et al., 2016
*Solanum lycopersicum*	Drought stress	Improved yield	Jokinen et al., 1999
*Triticum aestivum*	Cold stress	Increase in total osmolality, higher endogenous GB levels, induction of *wcor410* and *wcor413* genes, improved tolerance to photoinhibition of PSII	Allard et al., 1998
*Triticum aestivum*	Drought stress	Increased grain yield and higher number of grains per spike	Díaz-Zorita et al., 2001
*Triticum aestivum*	Drought stress	Improved STI (Stress tolerance index), enhanced levels of osmolytes (proline and GB), increased RWC	Gupta et al., 2014
*Triticum aestivum*	Drought stress	Higher net photosynthetic rate, higher maximal photochemistry efficiency of PSII, higher antioxidativeenzyme activities	Ma X.L. et al., 2006
*Triticum aestivum*	Drought stress	Increased spike length, higher number of spikelets per spike and of grains, improved yield, higher leaf turgor potential	Raza et al., 2014
*Triticum aestivum*	Drought stress	Stabilization of the function of the thylakoid membranes, suppression of chlorophyll degradation and enhancement of Ca^2+^-ATPase and Hill reaction activities, improved lipid composition of the thylakoid membranes	Zhao et al., 2007
*Triticum aestivum*	Salt stress	Higher endogenous GB levels, improved leaf water and osmotic potential, reduced Na^+^ and increased K^+^ and Ca^2+^, improved growth, enhanced activities of SOD, CAT, and POD	Raza et al., 2007
*Triticum aestivum*	Salt stress	Alleviated inhibition of photosynthesis	Rajasekaran et al., 1997
*Vigna unguiculata*	Salt stress	Increased total soluble sugar concentration and antioxidative enzymes (POD and PAL), increment of proline	Manaf, 2016
*Zea mays*	Cold stress	Prevention of chlorosis and reduced lipid peroxidation of the cell membranes	Chen et al., 2000
*Zea mays*	Drought stress	Increased height, leaf area and total dry weight	Reddy et al., 2013

Khan et al. (2014) showed that salicylic acid induced GB accumulation in *Vigna radiata* under salinity, with a consequent increase of glutathione, reduction of ethylene and oxidative stress, and improvement of photosynthesis. GB also protects photosynthesis by modifying the lipid composition of the thylakoid membranes in *Triticum aestivum* (Zhao et al., 2007). It increases soluble sugars and free amino acid accumulation to protect plant cells from salinity- and drought-induced osmotic stress in *Vigna unguiculata* (Manaf, 2016), *Phaseolus vulgaris* (Osman and Salim, 2016), and *Pisum sativum* (Osman, 2015). GB specifically increases its own content and improves the activity of antioxidant enzymes and metabolites, such as SOD, CAT, APX, proline and γ-amino butyric acid (GABA), reducing H_2_O_2_ and malondialdehyde (MDA) in *Prunus persica* (Shan et al., 2016), *Lolium perenne* (Hu et al., 2012), *Glycine max* (Malekzadeh, 2015), and *O. sativa* (Yao et al., 2016).

**FIGURE 2:**
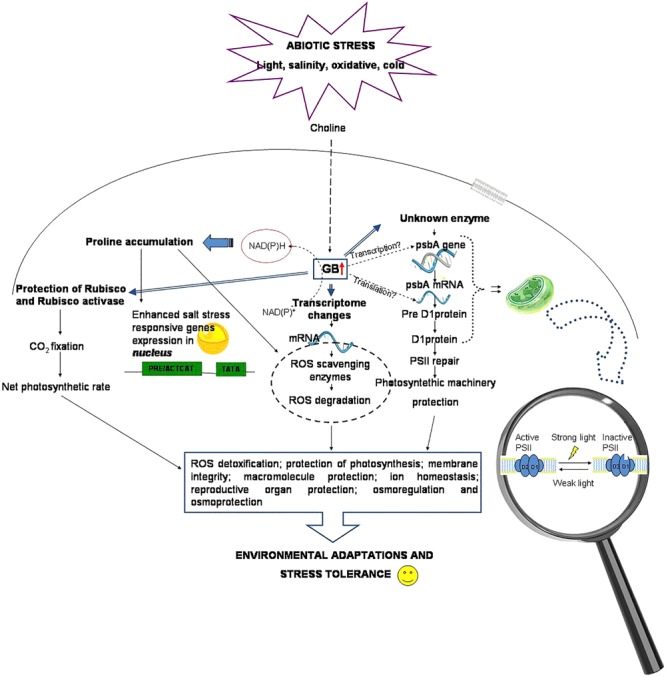
Glycine betaine mechanisms and protective roles via the ROS scavenging system.

DNA microarrays performed to study the gene expression modification induced by the application of exogenous GB (100 mM) to Arabidopsis leaves and roots, revealed that the expression of genes encoding enzymes involved in ROS scavenging, as well as gene encoding functions related to membrane trafficking (RabA4c GTPase) and to extracellular ferric reduction (FRO2 and FRO4), was prevalently enhanced. This evidenced the involvement of stress-induced ROS signaling in GB action (Einset and Connolly, 2009) (Figure 2).

However, the GB applied to roots is usually taken up and accumulated in the cytosol and only a small amount is translocated to chloroplasts, while, when applied to leaves, it is translocated to meristematic tissues, in particular flower buds and shoot apices, and then translocated to actively growing and expanding tissues (Mäkelä et al., 1996; Park et al., 2006). Ladyman et al. (1980) also found that after the application of GB to mature leaves of *H. vulgare* under water stress, osmolytes were translocated to the young expanding tissues. Therefore, in plants even if GB is applied to old or mature tissues, it reallocates to young actively growing tissues, where its protective functions are mainly required.

## Glycine Betaine Transport and Translocation

Although it is clear that GB endogenously accumulated or exogenously applied is reallocated in growing expanding tissues, knowledge on glycine transport and translocation remains fragmentary (Masood et al., 2016), and to date no specific transporters for GB have been reported in plants (Chen and Murata, 2011; Kumar et al., 2017).

The first direct demonstration of a GB transport activity was obtained by Schwacke et al. (1999) through the heterologous expression of a tomato gene, homologous to Arabidopsis proline trasporter *LeProT1* (Rentsch et al., 1996), in the yeast mutant 22574d. The latter was unable to grow on citrulline, proline, or GABA as the sole nitrogen source; however, when complemented with the LeProT1 protein, it was able to transport proline and GABA with a low affinity and GB with a high affinity. Breitkreuz et al. (1999) also found that the Arabidopsis GABA transporter ProT2 was strongly inhibited by GB, with a high affinity to the osmolytes. ProTs could therefore be considered general carriers, which allow the transport of compatible solutes, including GB, with stress protecting functions (Breitkreuz et al., 1999). However, Waditee et al. (2002) showed that in the betaine-accumulating mangrove, *Avicennia marina* under salinity *LeProT1* mRNA accumulated only in pollen; while in other tissues there was an increase of mRNA for GB/proline *A. marina* transporters 1, 2, and 3 (*AmT1, -2*, and *-3*). *AmT1* and *-2* were able to complement salt-sensitive GB and a proline-deficient *E. coli* mutant. Moreover, the main accumulation of *AmT1* under salinity was correlated to a major role for the transport of GB under osmotic stress. Subsequently, a gene homologous to *AmT1, BvBet/ProT1*, was isolated in *Beta vulgaris* by Yamada et al. (2009). A fusion protein GFP-BvBet/ProT1 was used to show the plasma membrane localization of the protein. In addition, both under control and salt-stress conditions, higher levels of *CMO* and *BvBet/ProT1* mRNA were found in older leaves than in young leaves, further demonstrating that GB is mainly synthesized in older tissues and then translocated to young expanding ones (Yamada et al., 2009). *In situ* hybridization experiments demonstrated that BvBet/ProT1 was localized in phloem and xylem parenchyma cells (Yamada et al., 2011). A comparison between Bet/ProTs from non-accumulating (*A. thaliana* Col-O) and GB accumulating (*B. vulgaris, Amaranthus tricolor*, and *Atriplex gmelinii*) plants expressed in a yeast mutant deficient for uptake of proline and GB, showed that all the transporters had lower Km and therefore a higher affinity for GB than proline. The uptake of both osmolytes was pH-dependent, with GB uptake at a higher rate by BvBet/ProT1 when the pH decreased to 4.5 and underwent an inhibition by the proton uncoupler carbonylcyanide m-chlorophenylhydrazone (CCCP). The same transporters exhibited a higher affinity for choline uptake rather than GB, particularly at higher pH (6.5), and were less dependent on the inhibitor CCCP, suggesting that Pro/BetTs enacts a symport mechanism for GB/H^+^ and choline/H^+^ with a different mechanism of proton binding (Yamada et al., 2011).

Tsutsumi et al. (2015), which localized CMO exclusively in mature leaves of *A. gmelinii*, found that in the same plants the *BetT* gene was expressed in bladder and stalk cells, in meso-phyll cells of young leaf laminae and in vascular tissues. This finding is in agreement with the translocation experiments of ^14^C-labeled GB in *H. vulgare* (Ladyman et al., 1980), *Brassica rapa* ssp. oleifera, *Glycine max, Pisum sativum, Lycopersicon esculentum*, and *T. aestivum* (Mäkelä et al., 1996) which suggested a long-distance translocation of GB, together with photosynthetic assimilates, via phloem, and the phloem localization of BvBet/ProT1 found by Yamada et al. (2011). Moreover, Park et al. (2006) demonstrated that when GB was applied to single mature leaves of tomato and accumulated in them, soon after, a large part of it was translocated to meristematic tissues, such as flower buds and shoot apices. In Arabidopsis (Sulpice et al., 2003) and tomato (Park et al., 2007) GB-accumulating transgenic plants also translocated GB, via phloem, actively accumulating it in growing flower buds and shoot apices.

Tsutsumi et al. (2015) suggested that one possible explanation for why GB is firstly synthetized in expanded tissues and then transported to young expanding ones, is that for its synthesis it is necessary to reduce ferredoxin, which is primarily produced by mature leaves.

## Glycine Betaine Role in Abiotic Stress Tolerance in Plants

Glycine betaine is one of the main compatible compounds present in Poaceae and Chenopodiaceae under salinity, and which is also involved in many other protective mechanisms against stress-related plant disorders (Ashraf and Foolad, 2007; Chen and Murata, 2008; Banu et al., 2010; Carillo et al., 2011; Khan et al., 2012). GB is an amphoteric metabolite highly soluble in water, and electrically neutral over a vast range of pH values (D’Amelia et al., 2018). The cellular concentration of GB, proline, or both, contribute to the osmotic pressure as a whole in many halophyte plants (Flowers et al., 1977). In glycophytes, GB is present at much lower levels than in halophytes. However, since it is compartmentalized solely to the cytosol and hyaloplasmic organelles, which account for about 20% of the volume of the cell or less, it is able to significantly contribute to the increase of osmotic pressure and can balance the vacuolar osmotic potential (Ashraf and Foolad, 2007; Cuin et al., 2009; Carillo et al., 2019). GB does not only act as an osmolyte for osmotic adjustment, but, as a zwitterion, it can interact with both hydrophilic and hydrophobic domains of protein complexes and membranes: this contributes to stabilizing and maintaining the structural and functional integrity of these molecules, protecting them from the detrimental effects of highly reactive oxygen species (ROS) (Sharma and Dietz, 2006; Ashraf and Foolad, 2007; Chen and Murata, 2008; Islam et al., 2009; Banu et al., 2010; Gupta and Huang, 2014). GB can reduce the salt-induced potassium efflux by regulating ion channels (Wei et al., 2017), and enhancing the enzymatic activity of plasma membrane H^+^-ATPase, increasing the phosphate uptake and regulating the phosphate homeostasis (Li et al., 2019). Furthermore, it is able to preserve the thermodynamic stability of macromolecules, reversing protein misfolding and/or aggregation without compromising their native functional activities (Khan et al., 2010). When GB is present at high levels, together with proline, it is so efficient in protecting plants by oxidative stress that antioxidant metabolites and enzymes play a minor role in ROS protection under salinity (Carillo et al., 2011; Annunziata et al., 2017; Woodrow et al., 2017).

Several beneficial effects of GB are summarized in Figure 2 and Table 2.

**Table 2:** Effect of endogenous glycine betaine under abiotic stress conditions.

Crop	Abiotic stress	Effect of endogenous GB under abiotic stress conditions	Reference
*Amaranthus tricolor*	Salt stress	Osmotic adaptation to salinity	Wang and Nii, 2000
*Beta vulgaris*	Salt stress	Maintenance of the intra-cellular osmotic balance between the cytoplasm and Na^+^ in the vacuole, protection of cytosolic enzymes from Na^+^ toxicity	Subbarao et al., 2001
*Beta vulgaris*	Water stress	Osmotic adjustment	Chołuj et al., 2008
*Hordeum maritimum*	Salt stress	Osmotic balance and protection of leaves from oxidative stress during the first phases of salt stress	Ferchichi et al., 2018
*Hordeum vulgare*	Cold stress	Improved survival of leaf laminae	Kishitani et al., 1994
*Spinacia oleracea*	Salt stress	Control of cellular osmotic potential	Di Martino et al., 2003
*Morus alba*	Salt stress	Osmotic adjustment	Agastian et al., 2000
*Oryza sativa*	Drought stress	Maintenance of RWC and GSH/GSSG ratio, lower reduction of K^+^, Ca^2+^, and Mg^2+^ content	Basu et al., 2010
*Prosopis alba*	Salt stress	Osmotic adjustment	Meloni et al., 2004
*Spinacia oleracea*	Salt stress	Protection of the oxygen-evolving Photosystem II complex	Papageorgiou et al., 1991
*Spinacia oleracea*	Salt stress	Osmotic adjustment and maintenance of photosynthetic capacity	Robinson and Jones, 1986
*Triticum aestivum*	Cold stress	Protection of plasma membrane	Zhang et al., 2010
*Triticum aestivum* L. cv. Glenlea	Freezing stress	Increased freezing tolerance	Allard et al., 1998
*Triticum aestivum*	Salt stress	Higher RWC and higher activity of antioxidant enzymes such as SOD, GR, and CAT	Sairam et al., 2002
*Triticum durum*	Salt stress	Function as osmolyte to balance water potential within root and shoot tissues	Carillo et al., 2005
*Triticum durum*	Salt stress	Protection of photosynthesis, increased nitrogen metabolism enzyme activities and ROS scavenging in young leaf tissues	Carillo et al., 2008, 2011
*Triticum durum*	Salt stress	Osmotic adjustment of root tissues of plants grown under low nitrate and salinity	Annunziata et al., 2017

**FIGURE 3:**
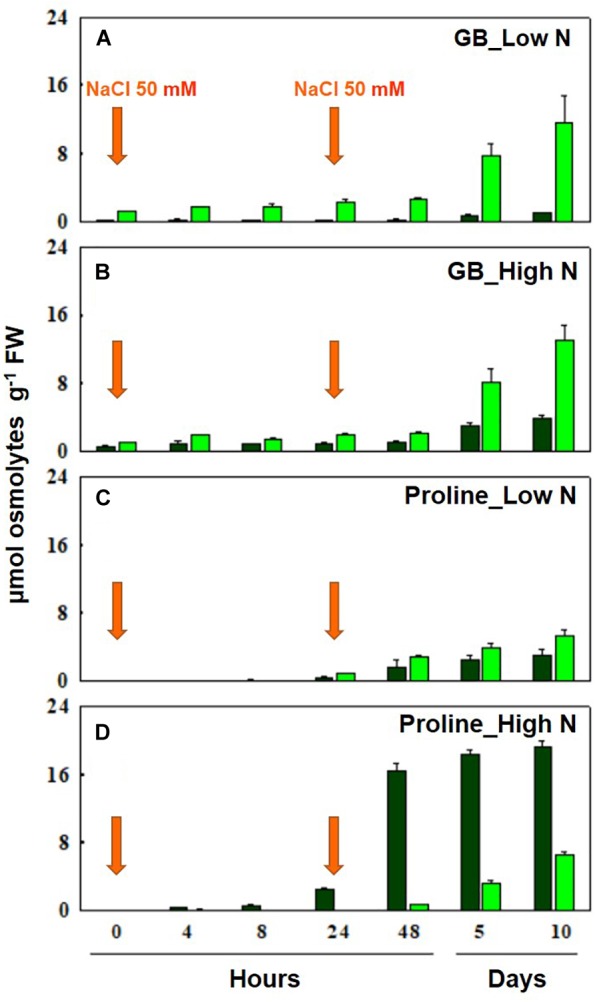
Accumulation of GB at low **(A)** and high **(B)** N, and proline at low **(C)** and high **(D)** N leaves of durum wheat plants after 10 days of hydroponic culture (0 h) and after 4, 8, 24, 48 h, 5 and 10 days of salt treatment. NaCl 50 mM was added twice at 0 and 24 h. Bar colors show older/mature (dark green) and younger (light green) tissues. Nitrate 0.1 (low N) or 10 mM (high N) was added on day 5 of hydroponic culture. The values are mean ± SD (*n* = 4) (data from Carillo et al., 2008, 2011).

It has been proven that the synthesis of GB is ontogenetically controlled in several plant species (Table 3). However, in bread wheat and durum wheat, it is asynchronous compared to that of proline and independent of nitrogen nutrition (Colmer et al., 1995; Carillo et al., 2008, 2011). In fact, GB is synthetized and accumulated in young leaf tissues during prolonged stress, and, as a quaternary nitrogen compound, its synthesis is independent of nitrate nutrition. Whereas, proline is accumulated more rapidly at the onset of stress and primarily in older leaves dependent on high nitrate (Carillo et al., 2008) (Figure 3). The lack of nitrogen nutrition influence on GB synthesis and accumulation, implies that nitrogen reserves within the plant can be employed to fulfill the metabolic demands of osmolytes, resulting from salt stress (Carillo, 2018). Therefore, GB and soluble sugars, like sucrose, but not proline, can play a major role in a plants adaptation to salinity under low nitrogen treatments; while proline is promptly synthetized, also in young tissues, under high nitrogen supply (Annunziata et al., 2017). However, GB and proline levels are highly correlated under salinity conditions, and their sum is equal in young expanding tissues of both high- and low-nitrogen grown plants. The presence of interchangeable levels of both compounds in young tissues, independent of nitrogen nutrition, would imply that resources are allocated in growing tissues in order to support and protect young growing tissues (Carillo et al., 2008). The fact that the presence of one of these metabolites limits that of the other could also be due to the proposed GB-dependent inhibition of proline accumulation (Gibon et al., 1997; Sulpice et al., 1998). However, the data of Carillo et al. (2008) conflict with this assumption since they showed that the synthesis and accumulation of proline antedate those of GB, and that the use of an inhibitor of proline synthesis, hydroxyl-proline, decreases GB accumulation.

**Table 3:** Spatial accumulation of endogenous glycine betaine.

Plant species and age	Stress	Glycine betaine (μmol g^-1^ FW)			Reference
		Old leaf tissues	Young leaf tissues	Roots	
*Amaranthus tricolor*	Control and NaCl 300 mM	Higher	Lower	Low	Wang and Nii, 2000
*Beta vulgaris* (1–1.5 m)	Control	10	20	4.2	Yamada et al., 2009
	NaCl 300 mM	40	120–125	16	
	Control and NaCl 300 mM	Higher *BvBet/ProT*1 mRNA levels			
*Gossypiumherbaceum* MI8	NaCl 200 mM	23.2	8.2–16.9		Gorham, 1996
*Hordeum vulgare* (21–26 days)	Control	0.3	0.3		Nakamura et al., 1996
	NaCl 200 mM	2.5–5	7.4–9.5		
*Hordeum vulgare* (21 days)	NaCl 200 mM	CMO expression level increased			Mitsuya et al., 2013
*Triticumaestivum* L. cv. Chinese Spring (CS) (18 days)	ControlNaCl 200 mM	0.94.4	6.617.5		Colmer et al., 1995
*Triticumaestivum* CS *X L. elongatum* am. (18 days)	ControlNaCl 200 mM	2.610.2	6.035.3		
*Triticum durum* (20 days)	NaCl 100 mM + 0.1 mM NO_3_	1.0	4.4	0.2	Carillo et al., 2008
	10 mM NO_3_	1.2	11.6	0.7	
	NaCl 100 mM + 10 mM NO_3_	4.0	5.9	0.6	
	Control	2.9	13.1	4.3	
*Zea mais L.* ibrids GB accumulators (4.5 week)	ControlNaCl 150 mM	0.01–0.030.02–0.14	0.01–0.70.02–4.1	0.010.01	Rhodes et al., 1989

Rhodes and Hanson (1993) found that plants do not show a significant GB breakdown. Therefore, the low levels of GB present in older plant tissues depend on a dilution mechanism that occurs via GB translocation from fully expanded to young growing tissues, since the latter is more prone to stress, as reported in oilseed rape, turnip rape, bread, and durum wheat (Maas and Poss, 1989; Mäkelä et al., 1996; Carillo et al., 2008). Annunziata et al. (2017) also found that the contribution of GB to osmotic adjustment in younger tissues is much higher than that in older tissues, independently of nitrogen nutrition. Even the expression level of CMO (Mitsuya et al., 2013) and BADH (Hattori et al., 2009) proteins was preferentially induced in younger leaves in barley plants under salinity. Since GB is accumulated only during prolonged stresses, and it cannot be metabolized, even if easily and efficiently transported from older to younger plant tissues, it has been supposed that it can play a pivotal role in protecting against salt stress young leaf and root tissues (Carillo et al., 2008, 2011). In view of this, Annunziata et al. (2017) ascribed the arrest of growth and differentiation of root tips of durum wheat under salinity to the delay in the synthesis of GB, which did not allow for the prompt contrast of the cytotoxic effect of the NaCl ions.

Carillo (2018) suggested that in young leaves of durum wheat plants, under high salinity the salt induced stomata closure restricts CO_2_ exchange and, consequently, reduces the RUBISCO CO_2_-fixation activity, while increasing the over-excitation of the photosynthetic apparatus and the production of ROS. In this condition, GB synthesis is induced to increase the protection of the photosynthetic apparatus (Chen and Murata, 2011; Kurepin et al., 2015).

Nevertheless, Carillo et al. (2011) showed that the relevance of GBs synthesis in durum wheat, is almost completely inhibited by high light (HL) even in the presence of high concentrations of NaCl. Woodrow et al. (2017) proved that in these plants, in which GB was not accumulated, the fine metabolic regulation of few specific primary metabolites, such as GABA, amides, minor amino acids and hexoses, could play a key role in the plants response to simultaneous stresses. The positive effect of GABA can be ascribed to its proton consuming synthesis that allows for the control of pH, and its nature of zwitterion which permits its accumulation in cytosol, where it acts as an osmolyte and ROS scavenger, without toxic effects. However, a possible more relevant effect is that its synthesis, operated by the glutamate decarboxylase (GAD), releases CO_2_ that can be used for RUBISCO and the simultaneous dissipation of excess energy produced by photosynthesis under HL and salinity. This re-start of the Calvin cycle reduces the pressure on the photosynthetic electron chain and decreases ROS production and photodamage (Carillo, 2018, and references therein).

## Conclusion

Metabolic engineering approaches and exogenous applications, aimed at increasing the synthesis and/or accumulation of GB in plants tissues, have been associated with the improvement in growth and survival of plants, ROS scavenging, osmoregulation of the cytosolic compartments, membrane stabilization, buffering of redox potential and induction of stress responsive genes that counteract the metabolism dysfunctions caused by stress. However, the efficacy of GB metabolism transformation for plant crops, cultured in field, has not been fully demonstrated. This might be because even if the GB concentration in transformed plants is significantly increased, it is still lower than that of a natural high accumulator species. Moreover, even if exogenous applications of GB that is targeted to the older damaged tissues has been tested, GB is promptly re-translocated to younger expanding tissues, where its protective functions are likely most required.

However, what is certainly clear is that in addition to this spatial discrepancy, that is the accumulation or re-allocation to young tissues after exogenous application, the synthesis of GB is also temporally delayed compared to other important osmolytes, such as proline. This most likely happens because GB cannot be metabolized. It is synthesized and accumulated only during extended stress, particularly in young tissues, as well as at low N nutrition. For this reason, it has been supposed that it plays a pivotal role in protecting young expanding tissues.

## Author Contributions

All authors listed have made a substantial, direct and intellectual contribution to the work, and approved it for publication.

## Conflict of Interest Statement

The authors declare that the research was conducted in the absence of any commercial or financial relationships that could be construed as a potential conflict of interest.
